# Efficacy and safety of spironolactone for the treatment of patients with acute heart failure: A protocol for systematic review

**DOI:** 10.1097/MD.0000000000022590

**Published:** 2020-10-23

**Authors:** Yan-lin Feng, Min Lu

**Affiliations:** a Department of Geriatrics, Yan’an People's Hospital, Yan’an; b Department of Cardiology, School Hospital of Xi’an International Studies University, Xi’an, Shaanxi, China.

**Keywords:** acute heart failure, efficacy, safety, spironolactone

## Abstract

**Background:**

This study will investigate the efficacy and safety of spironolactone for the treatment of acute heart failure (AHF).

**Methods:**

The following electronic databases will be retrieved in PUBMED, EMBASE, Cochrane Library, Web of Science, CINAHL, CBM, CNKI, and VIP database from inception through present. Two researchers will independently screen and assess the obtained literatures and extract outcome data. All study methodological quality will be assessed using Cochrane risk of bias tool, and all statistical analysis will be performed by RevMan 5.3 software. Additionally, we will undertake a narrative synthesis if it is possible.

**Results:**

This study will sum-marize most recent evidence to investigate the efficacy and safety of spironolactone for the treatment of AHF.

**Conclusion:**

This study will seek to assess the efficacy and safety of spironolactone for treating AHF.

**Systematic review registration:**

INPLASY202070053.

## 1 Introduction

Acute heart failure (AHF) is a major public health issue.^[1,2]^ It is a characterized by a rapid onset or acute worsening of symptoms (dyspnoea, orthopnoea, lower limb swelling) and signs (elevated jugular venous pressure, pulmonary congestion).^[3–5]^ It is resulted from a structural and/or functional cardiac abnormality.^[6,7]^ It is reported that AHF is associated with more than 26 million hospitalizations annually around the world.^[8–10]^ Its annual mortality rate after AHF hospitalization is still about 20% to 30%, and there is still high potential risk of subsequent hospitalization.^[11]^

Management of AHF relies on rapid recognition of its symptoms and signs.^[12,13]^ Previous studies reported that spironolactone can benefit patients with AHF.^[14–24]^ However, no systematic review specifically assesses the efficacy and safety of spironolactone alone for the treatment of patients with AHF. Thus, this systematic review will investigate the efficacy and safety of spironolactone for the treatment of AHF.

## 2 Methods

### 2.1 Study registration

We registered this study through INPLASY202070053. We report it according to the Preferred Reporting Items for Systematic Reviews and Meta-Analysis Protocol statement guidelines.^[25]^

### 2.2 Ethics and dissemination

It is not necessary to supply ethical approval, since this study only harvest data from published study. We will publish this study on a peer-reviewed journal or a conference meeting.

### 2.3 Study eligibility criteria

#### 2.3.1 Types of studies

This study will identify all potential randomized controlled trials (RCTs) on efficacy and safety of spironolactone in treating AHF, in spite of language and publication time.

#### 2.3.2 Types of participants

All patients who were diagnosed as AHF will be included in this study, irrespective gender, age, economic status, and other information.

#### 2.3.3 Types of interventions

##### 2.3.3.1 Experimental interventions

We will include any forms of spironolactone in treating patients with AHF.

##### 2.3.3.2 Control interventions

We will consider any therapies in treating AHF. However, we will exclude combined treatments with spironolactone.

#### 2.3.4 Type of outcome measurements

Outcomes are all-cause mortality, clinical congestion score, urine output, weight change, quality of life, and safety.

### 2.4 Search strategy and data management

#### 2.4.1 Search strategy

This study will search all potential studies in PUBMED, EMBASE, Cochrane Library, Web of Science, CINAHL, CBM, CNKI, and VIP database from initial through present. A specific description of search strategy of PUBMED is built in Table 1. We will modify similar search strategies and will apply them to other electronic databases. Additional searches will be conducted from any associated sources, such as conference proceedings, thesis, dissertations, and reference lists of relevant reviews.

**Table 1:**
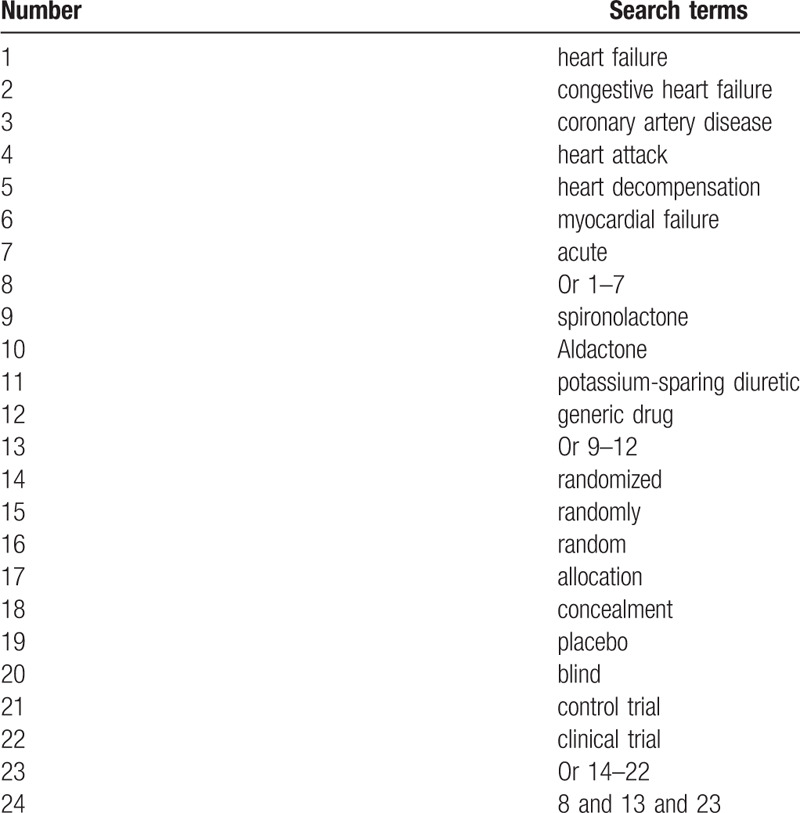
Specific description of search strategy of PUBMED.

#### 2.4.2 Study selection

We will examine titles/abstracts of searched studies, and will exclude all duplications and irrelevant studies. After eliminating those studies, we will check full papers of remaining potential studies based on all eligibility criteria. Two independent researchers will undertake selection of study. Disagreements between both of them will be figured out by another experienced researcher through discussion. We will present results of study selection in a flow diagram.

#### 2.4.3 Data extraction and management

Two researchers will independently extract main data from several aspects: author, time of publication, country, study design, study setting, sample size, gender, age, diagnostic criteria, inclusion and exclusion criteria, treatment details, comparators, frequency, dosage, outcomes, and adverse events. We will solve any disagreements between 2 researchers with the help of another researcher.

#### 2.4.4 Dealing with missing data

We will contact primary trial authors to request any insufficient, unclear or missing data. If we can not receive those data, we will analyze available data using intention-to-treat analysis.

### 2.5 Study quality assessment

Two researchers will appraise study quality using Cochrane risk of bias tool via 7 domains. If any different views occur, another experienced researcher will help to solve them through discussion.

### 2.6 Strategy for statistical analysis

This study will utilize RevMan 5.3 software to perform data analysis. The dichotomous data will be presented as risk ratio and 95% confidence intervals (CIs), and continuous data will be estimated as mean difference or standardized mean difference and 95% CIs. The statistical heterogeneity across eligible trials is examined by *I*^2^ test. If there is few heterogeneity (*I*^2^ ≤ 50%) among sufficient eligible studies on the same outcome, data will be pooled using a fixed-effect model, and meta-analysis will be performed. If there is obvious statistical heterogeneity across included studies (*I*^2^ > 50%), its sources will be identified using subgroup analysis. If we can not examine sources of obvious heterogeneity, we will perform descriptive analysis instead of meta-analysis.

### 2.7 Additional analysis

We will explore source of obvious heterogeneity based on the study information, patient characteristics, study quality and outcomes.

We will employ sensitivity analysis to test robustness of merged outcome results by excluding studies with high risk of bias.

We will examine reporting bias using funnel plot and Eggers regression test if at least 10 studies are included.^[26,27]^

## 3 Discussion

This is the first systematic review to yield high quality evidence on the efficacy and safety of spironolactone for the treatment of AHF. We will search both electronic databases and other literature sources to avoid missing potential studies. Two researchers will independently carry out study selection, study quality assessment, and data collection. Any division will be solved by a third researcher through discussion. The results of this study will inform helpful information to notify the management of spironolactone for AHF. Its findings may provide solid data and robust evidence of spironolactone for AHF for both clinical practice and patients.

## Author contributions

**Conceptualization:** Yan-lin Feng, Min Lu.

**Data curation:** Yan-lin Feng, Min Lu.

**Formal analysis:** Yan-lin Feng, Min Lu.

**Investigation:** Min Lu.

**Methodology:** Yan-lin Feng.

**Project administration:** Min Lu.

**Resources:** Yan-lin Feng.

**Software:** Yan-lin Feng.

**Supervision:** Min Lu.

**Validation:** Yan-lin Feng, Min Lu.

**Visualization:** Yan-lin Feng, Min Lu.

**Writing – original draft:** Yan-lin Feng, Min Lu.

**Writing – review & editing:** Yan-lin Feng, Min Lu.

## References

[1] Arrigo M Jessup M Mullens W Acute heart failure. Nat Rev Dis Primers 2020;6:16. 32139695 10.1038/s41572-020-0151-7 PMC7714436

[2] Čerlinskaitė K Javanainen T Cinotti R Acute heart failure management. Korean Circ J 2018;48:463–80. 29856141 10.4070/kcj.2018.0125 PMC5986746

[3] Arrigo M Ruschitzka F Flammer AJ Acute heart failure. Ther Umsch 2018;75:155–60. 30145979 10.1024/0040-5930/a000980

[4] Sinnenberg L Givertz MM Acute heart failure. Trends Cardiovasc Med 2020;30:104–12. 31006522 10.1016/j.tcm.2019.03.007

[5] Kurmani S Squire I Acute heart failure: definition, classification and epidemiology. Curr Heart Fail Rep 2017;14:385–92. 28785969 10.1007/s11897-017-0351-y PMC5597697

[6] Janssens U Acute heart failure. Med Klin Intensivmed Notfmed 2012;107:397–423. 22689257 10.1007/s00063-012-0118-x

[7] Mentz RJ O’Connor CM Pathophysiology and clinical evaluation of acute heart failure. Nat Rev Cardiol 2016;13:28–35. 26370473 10.1038/nrcardio.2015.134

[8] Ambrosy AP Fonarow GC Butler J The global health and economic burden of hospitalizations for heart failure: lessons learned from hospitalized heart failure registries. J Am Coll Cardiol 2014;63:1123–33. 24491689 10.1016/j.jacc.2013.11.053

[9] Solomon SD Dobson J Pocock S Candesartan in heart failure: assessment of reduction in M and morbidity I. Influence of nonfatal hospitalization for heart failure on subsequent mortality in patients with chronic heart failure. Circulation 2007;116:1482–7. 17724259 10.1161/CIRCULATIONAHA.107.696906

[10] Setoguchi S Stevenson LW Schneeweiss S Repeated hospitalizations predict mortality in the community population with heart failure. Am Heart J 2007;154:260–6. 17643574 10.1016/j.ahj.2007.01.041

[11] Chen J Normand SL Wang Y National and regional trends in heart failure hospitalization and mortality rates for Medicare beneficiaries, 1998-2008. JAMA 2011;306:1669–78. 22009099 10.1001/jama.2011.1474 PMC3688069

[12] Gouda P Ezekowitz JA Update on the diagnosis and management of acute heart failure. Curr Opin Cardiol 2019;34:202–6. 30547895 10.1097/HCO.0000000000000594

[13] Rayner-Hartley E Virani S Toma M Update on the management of acute heart failure. Curr Opin Cardiol 2018;33:225–31. 29194050 10.1097/HCO.0000000000000485

[14] Butler J Anstrom KJ Felker GM Efficacy and safety of spironolactone in acute heart failure: the ATHENA-HF randomized clinical trial. JAMA Cardiol 2017;2:950–8. 28700781 10.1001/jamacardio.2017.2198 PMC5675712

[15] Kapelios CJ Bonou M Vogiatzi P Association between high-dose spironolactone and decongestion in patients with acute heart failure: an observational retrospective study. Am J Cardiovasc Drugs 2018;18:415–22. 29971596 10.1007/s40256-018-0290-3

[16] Greene SJ Felker GM Giczewska A Spironolactone in acute heart failure patients with renal dysfunction and risk factors for diuretic resistance: from the ATHENA-HF trial. Can J Cardiol 2019;35:1097–105. 31230825 10.1016/j.cjca.2019.01.022 PMC6685766

[17] Bansal S Munoz K Brune S High-Dose spironolactone when patients with acute decompensated heart failure are resistant to loop diuretics: a pilot study. Ann Intern Med 2019;171:443–7. 31307058 10.7326/M18-3285

[18] Frederik HV Pieter M Koen A Spironolactone to increase natriuresis in congestive heart failure with cardiorenal syndrome. Acta Cardiol 2019;74:100–7. 29587582 10.1080/00015385.2018.1455947

[19] Oh J Kang SM Song MK Clinical benefit of spironolactone in patients with acute decompensated heart failure and severe renal dysfunction: data from the Korean Heart Failure Registry. Am Heart J 2015;169:713–20. 25965719 10.1016/j.ahj.2015.01.014

[20] Pedro FJ Mário S Carlos OJ Influence of spironolactone on matrix metalloproteinase-2 in acute decompensated heart failure. Arq Bras Cardiol 2015;104:308–14. 25993594 10.5935/abc.20140205 PMC4415867

[21] Ferreira JP Santos M Almeida S High-dose spironolactone changes renin and aldosterone levels in acutely decompensated heart failure. Cor et Vasa 2014;56:e463–70.

[22] Eng M Bansal S Use of natriuretic-doses of spironolactone for treatment of loop diuretic resistant acute decompensated heart failure. Int J Cardiol 2014;170:e68–9. 24268982 10.1016/j.ijcard.2013.11.023

[23] Lee KK Shilane D Hlatky MA Effectiveness and safety of spironolactone for systolic heart failure. Am J Cardiol 2013;112:1427–32. 24035170 10.1016/j.amjcard.2013.06.039

[24] Ma WD Shen YS Zhu CH Clinical application of spironolactone in acute myocardial infarction with heart failure. J Guangdong Colleg Pharm 2004;2:186–7.

[25] Shamseer L Moher D Clarke M PRISMA-P Group. Preferred reporting items for systematic review and meta-analysis protocols (PRISMA-P) 2015: elaboration and explanation. BMJ 2015;349:g7647. 10.1136/bmj.g7647 25555855

[26] Sutton AJ Duval SJ Tweedie RL Empirical assessment of effect of publication bias on meta-analyses. BMJ 2000;320:1574–7. 10845965 10.1136/bmj.320.7249.1574 PMC27401

[27] Egger M Davey Smith G Schneider M Bias in meta-analysis detected by a simple, graphical test. BMJ 1997;315:629–34. 9310563 10.1136/bmj.315.7109.629 PMC2127453

